# Analysis of bacterial and fungal communities in *Marcha* and *Thiat*, traditionally prepared amylolytic starters of India

**DOI:** 10.1038/s41598-017-11609-y

**Published:** 2017-09-08

**Authors:** Shankar Prasad Sha, Kunal Jani, Avinash Sharma, Anu Anupma, Pooja Pradhan, Yogesh Shouche, Jyoti Prakash Tamang

**Affiliations:** 1 DAILAB (DBT-AIST International Laboratory for Advanced Biomedicine), Bioinformatics Centre, Department of Microbiology, School of Life Sciences, Sikkim University, Gangtok, 737102 India; 2 National Centre for Microbial Resource, National Centre for Cell Science, Pune, 411021 India

## Abstract

*Marcha* and *thiat* are traditionally prepared amylolytic starters use for production of various ethnic alcoholic beverages in Sikkim and Meghalaya states in India. In the present study we have tried to investigate the bacterial and fungal community composition of *marcha* and *thiat* by using high throughput sequencing. Characterization of bacterial community depicts phylum *Proteobacteria* is the most dominant in both *marcha* (91.4%) and *thiat* (53.8%), followed by *Firmicutes*, *and Actinobacteria*. Estimates of fungal community composition showed *Ascomycota* as the dominant phylum. Presence of *Zygomycota* in *marcha* distinguishes it from the *thiat*. The results of NGS analysis revealed dominance of yeasts in *marcha* whereas molds out numbers in case of *thiat*. This is the first report on microbial communities of traditionally prepared amylolytic starters of India using high throughput sequencing.

## Introduction

Traditional practice of sub-culturing by back-sloping and preservation of essential native microbiota consisting of consortia of yeasts, molds and bacteria, in the form of dry, flattened, or round balls, for alcoholic beverages production in South-East Asia including the Himalayan regions of India, Nepal, Bhutan, and China is the worth wisdom of the ethnic people for centuries^1^. Some common and uncommon amylolytic starters in Asia are *marcha* of India, Nepal, and Bhutan, *hamei*, *humao*, *thiat*, *phab* of India, *men* of Vietnam, *bubod* of the Philippines, *chiu/chu* of China and Taiwan, *loogpang* of Thailand, *ragi* of Indonesia, *nuruk* of Korea, *mae/dombae/buh/puh*in Cambodia, etc.^2–7^ Traditionally prepared Asian amylolytic starters have consortia of mixed micrbiota representing filamentous molds, yeast and bacteria^1–3^, hence many researchers have studied the fungal, yeast and bacterial populations in Asian starter cultures, commonly based on culture-dependent techniques including phenotypic and 16S rRNA sequencing, and isolated and identified filamentous molds *Absidia corymbifera*, *Amylomyces rouxii*, *Botryobasidium subcoronatum*, *Mucor circinelloides* forma *circinelloides*, *Mucor hiemalis*, *Rhizopus oryzae*, *Rhi*. *microsporus*, *Rhi*. *chinensis*, and *Rhi*. *stolonifer*, *Xeromyces* bisporus^5, 8, 9^; yeasts *Candida glabrata*, *C*. *tropicalis*, *Clavispora lusitaniae*, *Issatchenkia* sp., *Pichia anomala*, *P*. *ranongensis*, *P*. *burtonii*, *Saccharomycopsis fibuligera*, *Sm*. *capsularis*, *Saccharomyces cerevisiae*, *Sacch*. *Bayanus* ^5, 9–13^; and bacteria *Acetobacter orientalis*, *A*. *pasteurianus*, *Bacillus amyloliquefaciens*, *B*. *circulans*, *B*. *sporothermodurans*, *B*. *subtilis*, *Pediococcus pentosaceus*, *Lactobacillus bifermentans*, *Lb*. *brevis*, *Lb*. *plantarum*, *Weissella confusa*, *W*. *paramesenteroides* ^5, 14–16^.

Introduction of culture-independent methods and it’s applicability in food microbiology^7, 17^, has been a motivation for few researchers to profile the microbial community structure of some Asian starter cultures using PCR-DGGE, pyrosequencing, etc. which is suggestive to provide more insight into the microbial diversity of ethnic starters^3, 5, 18–22^. Rapid evolution in next generation sequencing (NGS) technologies has enabled researchers to have increased accuracy, throughput, with reasonably low cost and in relatively short period of time^17, 23^. However, there are still a limited number of studies, characterizing the microbial community composition of fermented foods such as cheese^24–26^, kefir grains^27^, some ethnic Asian fermented foods^28–31^. Furthermore, the information on the community composition of Asian starter culture is rudimentary and needs in depth exploration using cutting edge technologies^7^.

**Figure 1:**
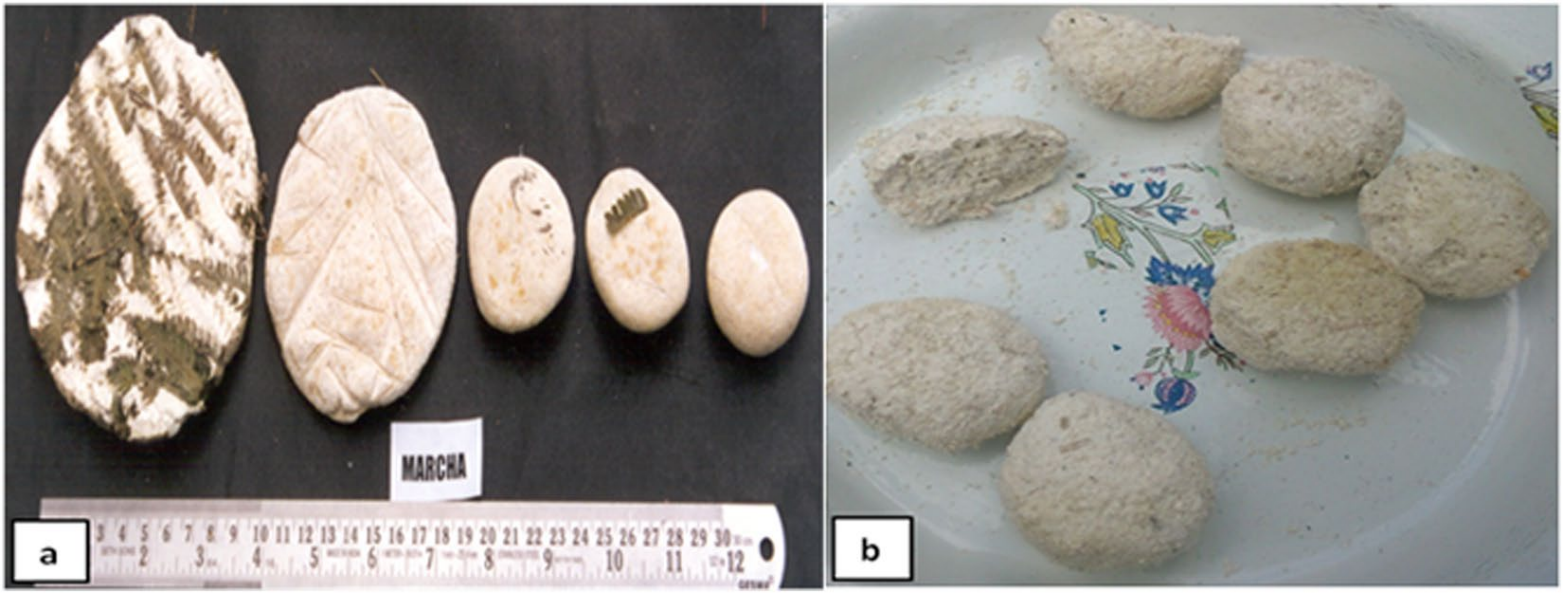
Traditionally prepared amylolytic starter cultures (**a**) *Marcha* and (**b**) *Thiat*.

In present study, we attempted to profile the microbial community composition of *marcha* and *thiat*, traditionally prepared ethnic starter cultures of India using targeted amplicon sequencing. We selected two different traditionally prepared amylolytic starter cultures from two regions in India, *marcha* (Fig. 1a) from Sikkim (www.sikkim.gov.in) and *thiat* (Fig. 1b) from Meghalaya (www.megtourism.gov.in). *Marcha* is prepared from soaked rice with some wild herbs ((*Plumbago zeylanica*, *Buddleja asiatica* and *Vernonia cinerea*), ginger and red dry chili, 1–2 % of previously prepared *marcha* powder as an inoculum, crushed in a wooden mortal by wooden pestle, mised and dough are made into round to flatted cakes of different size and shape. Cakes are covered with fern fronds (*Glaphylopteriolopsis erubeseens*), fermented at room temperature for 24 h, sun dried for 3–5 days and are used as amylolytic starters for production of cereal-based ethnic fermented beverages such as *kodo ko jaanr*, *bhaati jaanr*, *raksi*, etc.^2^ During *thiat* preparation, soaked glutinous rice is grinded with leaves and roots of wild plant *Amomum aromaticum*, 1–2% of old *thiat*, mixed and made into a dough by adding water. Flat to round balls are made and fermented for 1–3 days. The freshly prepared *thiat* balls are sun dried for 3–5 days. It is used to ferment alcoholic beverage locally called *kiad* in Meghalaya^2^. Fermentation process involved in preparation of these starters is unconditional and may harbor both bacterial and fungal communities as consortia. Therefore, we aimed to explore the bacterial and fungal (filamentous molds and yeasts) communities in *marcha* and *thiat*. This is the first report on complete microbial community profile of traditionally prepared amylolytic starters of India using NGS technique.

## Results

### Characterizing microbial diversity

High throughput sequencing and quality trimming of 16S rRNA and ITS gene yielded ~0.85and ~0.29 million quality reads in both *marcha* and *thiat*, respectively, which was used for subsequent analysis. Taxonomic assignment of sequences with the reference database resulted into 5,015 operational taxonomic units (OTUs). The average Good’s coverage of both the samples of *marcha* and *thiat* for 16Sr RNA amplicon sequencing was found to be 99.08% ± 0.1% (mean ± SD) whereas for ITS region was recorded as 87.5% ± 17.6% (mean ± SD) indicating majority of the diversity was captured.

The estimates of alpha diversity indices revealed significant differences between *marcha* and *thiat* when computed for both the bacterial and fungal diversity (Table 1a and b). The bacterial species richness was found to be higher in *thiat* (4256.83) than *marcha* (1520.92), in contrast, fungal species richness depicts higher in *marcha* (5.25) over *thiat* (5.0). Significant variations were also noticed in non-parametric shannon index for bacterial communities in *thiat* (5.48) and *marcha* (4.01). Shannon index for fungal communities follow the reverse trend with *marcha* (2.25) and *thiat* (1.80). This observation is suggestive of higher bacterial diversity in *thiat* while *march*a showed higher fungal diversity.

**Table 1:** Alpha diversity estimation.

	Chao1	Goods coverage	Shannon	Simpson
**a) Bacterial**				
*Marcha*	1520.925	0.998902539	4.01115959	0.866763863
*Thiat*	4256.838	0.997475969	5.489325073	0.940199394
**b) Fungal**				
*Marcha*	5.25	0.75	2.25	0.78125
*Thiat*	5	1	1.802366931	0.671398892

Non parametric alpha diversity was calculated for ethnic amylolytic starter cultures *marcha* and *thiat*.

### Bacterial community profile of *thiat* and *marcha*

**Figure 2:**
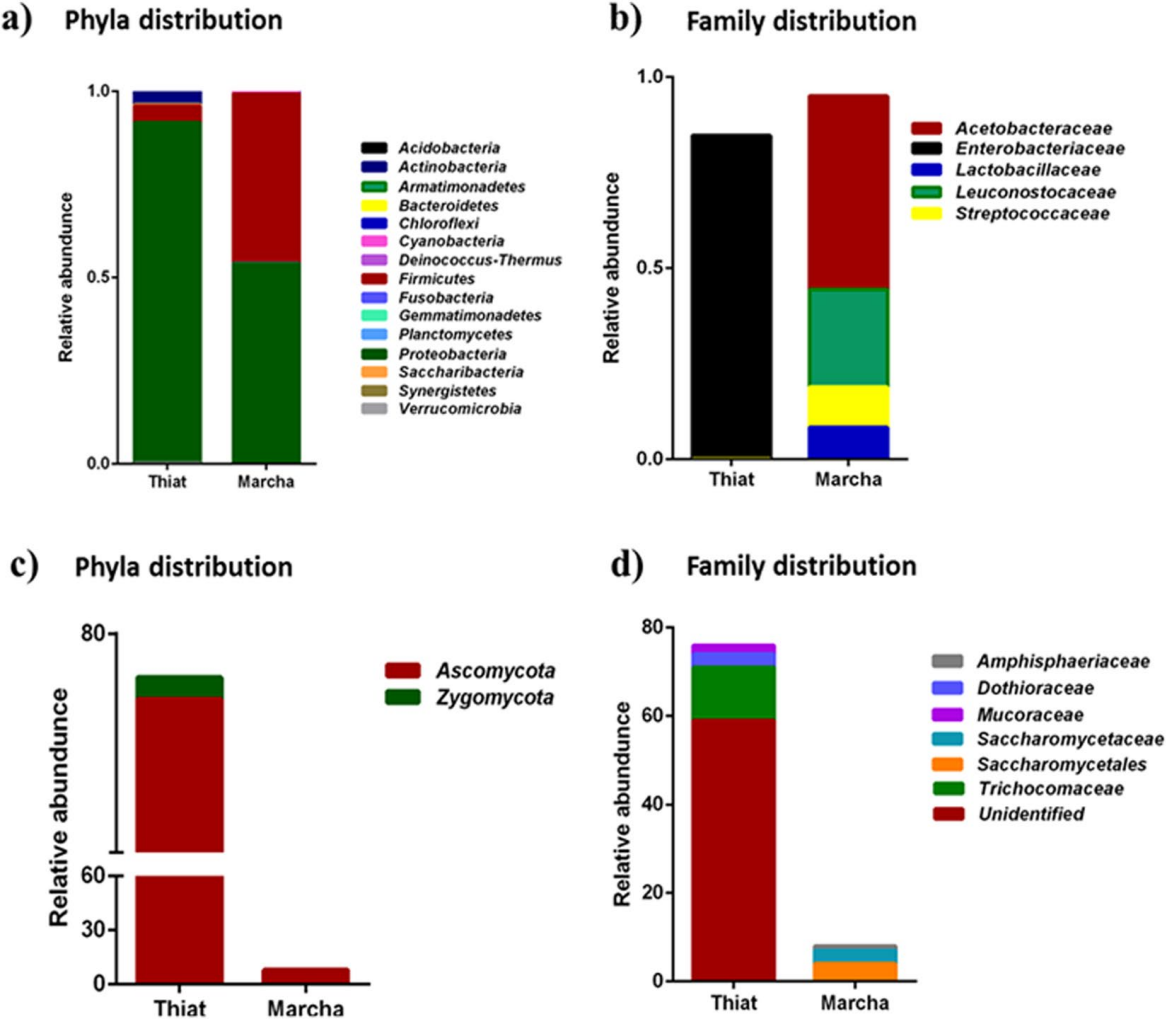
Taxa distributions of phylum and family at different phylogenetic level in *thiat* and *marcha*. (**a**) bacterial phyla; (**b**) bacterial family; (**c**), fungal phyla and (**d**) fungal family.

16S rRNA gene amplicon sequencing yielded 15 bacterial phyla in *thiat* and *marcha*, respectively (Fig. 2a). In *thiat* bacterial phyla distributions were *Proteobacteria* (91.4%), *Actinobacteria* (4%), *Firmicutes* (4%) and the rest (0.6%) constituted the minor phyla *Cyanobacteria*, *Bacteroidetes*, *Verrucomicrobia*, *Fusobacteria*, *Planctomycetes*, *Deinococcus-Thermus*, *Chloroflexi*, *Synergistetes*, *Acidobacteria*, *Saccharibacteria*, *Gemmatimonadetes*, *Armatimonadetes*. In *marcha* the phyla distributions of bacteria were *Proteobacteria* (53.8%), *Firmicutes* (45.4%) and other minor phyla were 0.8% constituting *Actinobacteria*, *Cyanobacteria*, *Bacteroidetes*, *Verrucomicrobia*, *Fusobacteria*, *Planctomycetes*, *Deinococcus-Thermus*, *Chloroflexi*, *Synergistetes*, *Acidobacteria*, *Saccharibacteria*, *Gemmatimonadetes*, and *Armatimonadetes*. The abundance of thirteen minor phyla was very less hence percentage of composition was not shown in Fig. 2a. Bacterial phylum *Proteobacteria* was found to outnumber other bacterial phyla in *thiat* whereas *marcha* was found to constitute *Proteobacteria* and *Firmicutes* as major phyla.

At family level, OTUs with ≥1% abundance were filtered which differed quantitatively between *thia*t and *marcha* (Fig. 2b). The family level distributions of bacteria in *thiat* were *Enterobacteriaceae* (84.6%), *Microbacteriaceae* (3.24%), *Enterococcaceae* (2.47%), *Clostridiaceae* (1.13%) *Neisseriaceae* (0.87%) and *Oxalobacteraceae* (0.59%) (Fig. 2b). Whereas the family level of bacterial distributions in *marcha* were *Acetobacteraceae* (50.6%), *Leuconostocaceae* (25.5%), *Streptococcaceae* (10.5%), *Lactobacillaceae* (8.38%), *Burkholderiaceae* (2.13%) and *Staphylococcaceae* (0.54%) (Fig. 2b).

**Figure 3:**
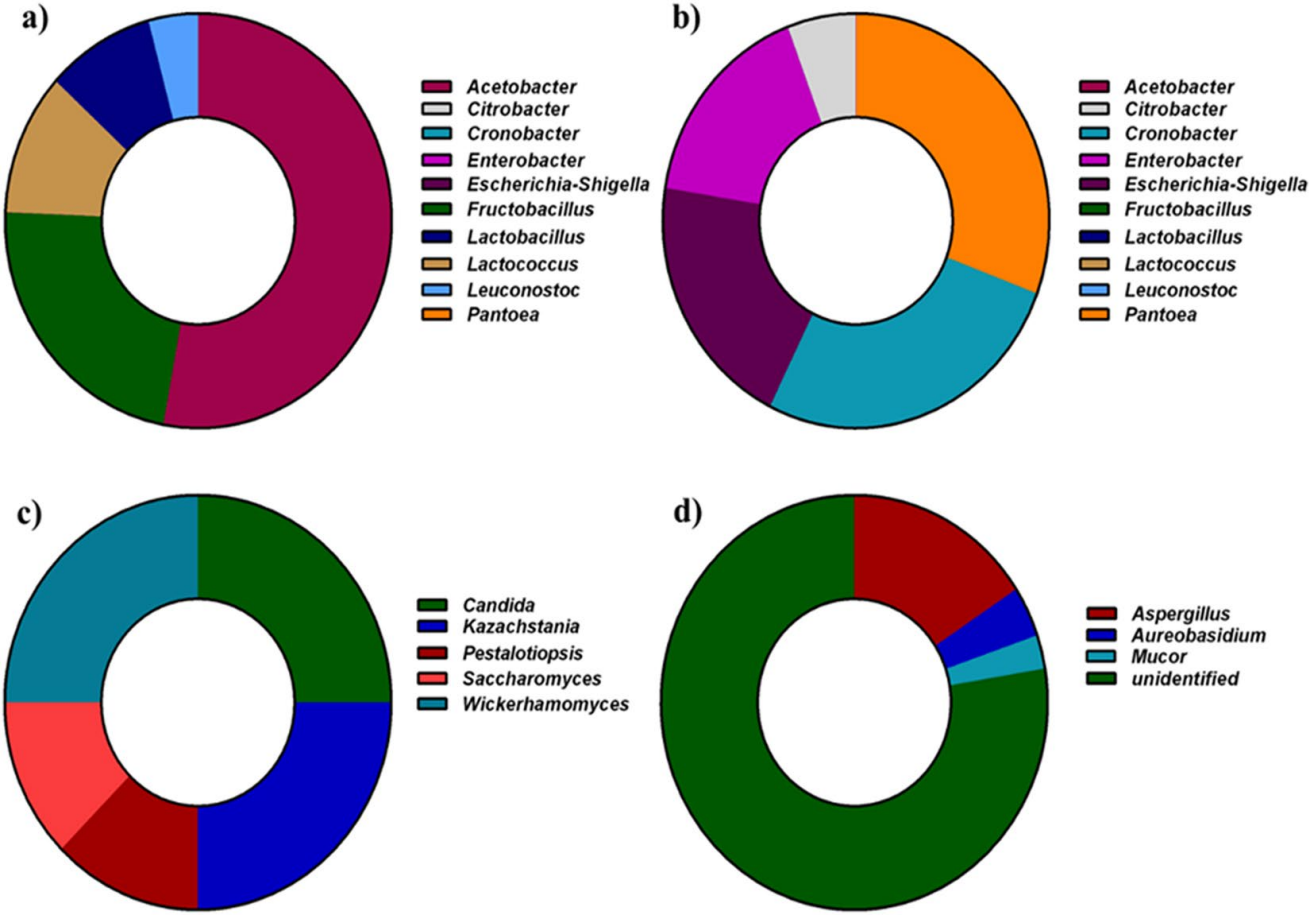
Taxa distributions of genus at different phylogenetic level. (**a**) bacterial genera in *marcha*; (**b**) bacterial genera in *thiat*; (**c**) fungal genera in *marcha* and (**d**) fungal genera in *thiat*.

At the genus level, OTUs with ≥1% abundance were filtered (Fig. 3a,b), which retained 18 differentially abundant genera in both samples of *marcha* and *thiat*. Distribution of bacterial genera in *marcha* were *Acetobacter* (52.6%), *Fructobacillus* (21.1%), *Lactococcus* (10.3%), *Lactobacillus* (8.4%), *Leuconostoc* (4.0%) (Fig. 3a), *Burkholderia* (2.1%) and *Gluconacetobacter* (1.4%). Genera in *thiat* were *Pantoea* (32.4%), *Cronobacter* (21.4%), *Escherichia*-*Shigella* (15.5%), *Enterobacter* (13.1%), *Citrobacter* (4.2%) (Fig. 3b), *Salmonella* (3.2%), *Serratia* (2.8%), *Enterococcus* (2.5%), *Curtobacterium* (2.2%), *Kluyvera* (1.6%) and *Clostridium* (1.1%). The composition percentage of bacterial genera which was less than 3.9% was not shown in Fig. 3a,b.

### Fungal (filamentous molds and yeasts) composition in *thiat* and *marcha*

Fungal ITS gene sequencing and taxonomic analysis demonstrated the predominance of yeast phylum *Ascomycota* (98.6%) in *thiat*, whereas the distribution of filamentous phyla *Zygomycota* was only 1.4% (Fig. 2c). However, in *marcha* only yeast phylum *Ascomycota* constituted the fungal diversity (Fig. 2c). Filamentous mold phylum was not detected in *marcha*. Distributions of fungi (filamentous molds and yeasts) at the family level in *thiat* were *Trichocomaceae* (15.7%), *Dothioraceae* (3.94%), *Mucoraceae* (2.63%) and unidentified fungi (77.73%). Whereas the distributions of yeasts at the order/family level in *marcha* were *Saccharomycetales* (50%), *Saccharomycetaceae* (37.5%) and *Amphisphaeriaceae* (12.5%). (Fig. 2d). Distributions of yeasts genera in *marcha* were *Wickerhamomyces* (25%), *Candida* (25%), *Kazachstania* (25%), *Saccharomyces* (12.5%) and *Pestalotiopsis* (12.5%) (Fig. 3c). The filamentous mold genera distribution in *thiat* were *Aspergillus* (15.7%), *Aureobasidium* (3.9%) and *Mucor* (2.7%) and unidentified genera (77.7%) (Fig. 3d). The unidentified genera represented the yeast phylum *Ascomycota* in *thiat*. The sequence reads showed the species of filamentous molds were *Aspergillus penicillioides*, *Aureobasidium pullulans* and *Mucor circinelloides*, whereas the yeasts species were *Wickerhamomyces anomalus*, *Candida quercitrus* and *Kazachstania exigua* (data not shown).

## Discussion

Our study provides comprehensive microbial diversity analysis using deep sequencing approach of ethnic amylolytic starter from India. Quantitative differences were noted for the presence of bacterial and fungal taxa among *marcha* and *thiat*; which could be the consequence of differences in the preparation, incubation period and most importantly the type of preservations. Alpha diversity estimation of amylolytic starters *marcha* and *thiat* using species richness and non-parametric Shannon index suggested higher bacterial diversity in *thiat* while *marcha* shows the higher assemblage of fungal diversity with dominance of yeast phylum *Ascomycota*. Persistence of higher fungal diversity in *marcha* is determinant factor suggesting the higher acidic conditions of *marcha*; in contrast, higher bacterial diversity of *thiat* depicts the faster turnover from acidic to alkali with the presence of acid neutralizing bacterial taxa^32^.

*Acetobacter*, *Fructobacillus*, *Lactococcus*, *Lactobacillus*, *Leuconostoc*, *Burkholderia*, and *Gluconacetobacter* were the predominant bacterial genera in *marcha*. Higher proportion of *Acetobacter* was possibly due to its retention and enrichment during fermentation. We observed relatively lower proportion of *Streptococcus* and *Lactococcus* than *Lactobacilli;* as *Lactobacilli* have high acid tolerance over former two^33^. Though some species of *Lactococcus* have low acid tolerance, however, they could be isolated from raw milk and were found flourishing during the early stage of fermentation^24^. This supports the lower abundance of *Lactococcus* than *Lactobacillus* as seen in our samples. Another interesting observation was absence of *Pediococcus* in bacterial community profile which was otherwise present as a one of the dominant genus in earlier report by culture dependent methods in *marcha* ^10, 16^. Furthermore, since there is no earlier report on microbial composition based on culture dependent or culture-independent methods of *thiat* the present study describe microbial diversity of *thiat* using NGS method as its first report. *Pantoea*, *Cronobacter*, *Escherichia*, *Shigella*, *Enterobacter*, *Citrobacter*, *Salmonella*, *Serratia*, and *Enterococcus* depicts most dominant bacterial genera of *thiat* each comprised over 0.1% of total bacterial sequences. Significantly varied microbial composition among *thiat* and *marcha* is a clear indication of differences in amylolytic starters. Genus *Enterobacter* was also detected in Mexican alcoholic beverages speculated to originate from the bacterial contamination in raw milk and they subsequently decreased during the fermentation process^34^. The lactic acid bacterium such as *Lb*. *plantarum* seemed to be one factor for the good quality of the alcoholic beverages, as it can perform malolactic fermentation to decrease wine acidity^32^ and also produces bacteriocins^35^.

Exploration of fungal diversity of ethnic amylolytic starters suggested higher abundance of yeast in *marcha* and *thiat* constitutes for 32.33-fold yeast to the filamentous molds. This observation was in coherence with the earlier report of culture-dependent studies showing the dominance of *Mucor* and *Rhizopus* genera of *Mucorales* in *marcha* ^8^. Interestingly no filamentous molds were detected in *marcha* using the applied high throughout sequencing method; the exact reasons for the observed variation in the microbiota have not been identified. This may be due to lower abundance of molds, limited sample size and/or age of the sample and finally also due to inadequate cell lysis which may prevent the release of nucleases^36^. Our study was in accordance to the previous reports describing the exposure of cheese to different external environments such as manufacturing process; geographical region, etc have varied impact on the microbial composition of the end products^28^. Thus, we speculate that the factor of geographic environment including altitudes and climate play a more significant role over the manufacturing process in resulting in the different microbial compositions of the starter culture under study. Some other crucial factors that may affect the composition of microbial communities in fermented amylolytic starters are level of hygiene, quality of the glutinous rice, water, as well as the back slopping technique. In this study three dominant yeasts in *marcha* were *Wickerhamomyces anomalus*, *Candida quercitrus* and *Kazachstania exigua*, followed by *Saccharomyces* and *Pestalotiopsis* which also accompany the findings of ref. 21 by PCR-DGGE method. ITS gene sequences analysis of the *thiat* revealed the existence of *Aspergillus penicillioides*, *Aureobasidium pullulans* and *Mucor circinelloides* as the most dominant filamentous molds in *thiat*. At family level *thiat* shows *Trichocomaceae*, *Dothioraceae*and *Mucoraceae*as the major constituents of fungal community composition emphasizing the significant differences between *thiat* and *marcha* viz differences in starter substrates, preparations, inoculums, consortia, geography, hygiene, preservation technique, caloric values etc.

In the present study *Ascomycota* was dominant in starter cultures of India like in Korean and Chinese starters cultures, which was also reported earlier, based on NGS tools, in Korean alcoholic beverages^3^ and in Chinese liquors^37^. We could also expect similar observation in case of *marcha* as it has higher abundance of lactic acid bacteria. *Aspergullus oryzae* has strong secretion of amylases including alpha-amylase, which may accelerate the degradation of grains and provide more nutrients for microbes in alcoholic fermentation^38^.

Amylolytic starter culture-making technology reflects the traditional knowledge of the ethnic Indian people on sub-culturing desirable inocula from previous batch to new culture using rice as base substrates by back-sloping method. This technique preserves the consortia of microbial community ranging from filamentous molds, yeasts and bacteria which were co-existed in traditionally prepared amylolytic and alcohol producing starters^7^, and also preserves vast biological genetic resources, otherwise, which may be forced to disappear. Fermented beverages produced by using amylolytic starters in India are generally mild-alcoholic (4–5%), sweet taste with several health benefits to the local consumers as high source of calories, some vitamins and minerals^2^. Ethnic fermented beverages and alcoholic drinks have the potential to grow beverage industry if proper scientific and technical support are applied to the existing indigenous practices of home based alcoholic fermentation.

## Materials and Methods

### Sample collection

Samples of sun-dried amylolytic starters *marcha* and *thiat* were collected immediately after the preparation from local people of Gangtok and Shillong in Sikkim and Meghalaya states of India, respectively. Dry samples were transferred to sterile containers, sealed, and stored at desiccator at room temperature for the further analysis.

### Community DNA Extraction

The total community DNA was extracted using ProMega DNA kit (ProMega). 1g of amylolytic starter culture sample was suspended in lysis solution and incubated at 65 °C for 15 min. Subsequently, the RNA was eliminated from the cellular lysate by administering the RNase solution following incubation at 35 °C for 15 min. The residual proteins were removed by adding protein precipitation solution and centrifuged at max speed. Finally, the DNA was precipitated by adding isopropanol, which was purified with two washes of 70% ethanol. The quality of DNA was checked on 0.8% agarose gel and concentration was measured using Nano-DropND-1000 spectrophotometer (Nano Drop technologies, Willington, USA) as described by ref. 39. The DNA was stored at −20 °C until further processing.

### Amplicon sequencing

Amongst the nine hypervariable regions of bacterial 16S rRNA gene, we have targeted V4 hyper-variable region^40^ to investigate bacterial diversity of *marcha* and *thiat*. The universal 16S rRNA gene primer sets F515 and 806R^41^ was used for the amplification of V4 hyper-variable region. Similarly, fungal Internal Transcribed Spacer (ITS) II region was targeted for taxonomic profiling amylolytic starters, which was subjected to amplification using ITS1 and ITS2 primers. The library preparation of both the 16S rRNA and ITS gene amplicons were in accordance with the protocols of Illumina (USA). These amplicon libraries were further processed for sequencing using AMPure XT beads (Beckman Coulter Genomics, Danvers, MA, USA). The resultant product was screened with the LabChip GX (Perkin Elmer, Waltham, MA, USA) and with the Library Quantification Kit for Illumina (Kapa Biosciences, Woburn, MA, USA). Subsequently, the 16S rRNA and ITS gene library were sequenced on the Illumina MiSeq platform using 2x 250bp chemistry. The sequences obtained from high throughput sequencing effort were submitted to National Center for Biotechnology Information (NCBI) which are available under BioProject ID PRJNA376467.

### Bioinformatics analysis

The raw sequences generated from MiSeq platform was assembled using FLASH tool (Fast Length Adjustment of Short reads) a Paired end assembler for DNA sequences^42^. The assembled reads were subjected to quality filtering using via Quantitative Insights into Microbial Ecology (QIIME) 1.8^42^. Sequence reads were assigned to bacterial and fungal operational taxonomic units (OTUs) by a closed reference-based OTU picking approach by using SILVA and UNITE reference databases, respectively. The OTU picking was carried out using UCLUST method with similarity threshold of 97%^43^. Taxonomic assignments were performed using RDP naïve bayesian classifier^44^. Alpha diversity indices like Chao, Shannon and Simpson were calculated via QIIME after rarefying all samples to the same sequencing depth^45, 46^.

### Data availability

The sequences obtained from high throughput sequencing effort, was submitted to NCBI which are available under Bio Project ID PRJNA376467.
